# The Efficacy of Entomopathogenic Nematodes Plus an Adjuvant against *Helicoverpa zea* and *Chrysodeixis includens* in Aboveground Applications

**DOI:** 10.2478/jofnem-2024-0018

**Published:** 2024-05-08

**Authors:** Minling Zhang, Nathan Spaulding, Gadi V.P. Reddy, David I Shapiro-Ilan

**Affiliations:** USDA-ARS, Southern Insect Management Research Unit, Stoneville, MS 38776, USA; USDA-ARS, SE Fruit and Tree Nut Research Unit, Byron, GA 31008, USA

**Keywords:** *Helicoverpa zea*, *Chrysodeixis includens*, entomopathogenic nematode, adjuvant

## Abstract

In the southern United States, corn earworm, *Helicoverpa zea* (Boddie), and soybean looper, *Chrysodeixis includens* (Walker) are economically important crop pests. Although Bt crops initially provided effective control of target pests such as *H. zea*, many insect pests have developed resistance to these Bt crops. Alternative approaches are needed, including biological control agents such as entomopathogenic nematodes (EPNs). However, the effectiveness of EPNs for aboveground applications can be limited due to issues such as desiccation and ultraviolet radiation. Effective adjuvants are needed to overcome these problems. Ten strains of EPNs were tested for virulence against eggs, first to fourth instars, fifth instars, and pupae of *H. zea* and *C. includens* in the laboratory. These 10 EPN strains were *Heterorhabditis bacteriophora* (HP88 and VS strains), *H. floridensis* (K22 strain), Hgkesha (Kesha strain), *Steinernema carpocapsae* (All and Cxrd strains), *S. feltiae* (SN strain), *S. rarum* (17c+e strain), and *S. riobrave* (355 and 7–12 strains). EPNs could infect eggs of *H. zea* or *C. includens* in the laboratory, but the infection was low. The mortality caused by 10 EPN strains in seven days was significantly higher for the first to fourth instars of *H. zea* compared to the control, as was the fifth instars of *H. zea*. Similarly, for the first to fourth and fifth instars of *C. includens*, the mortality was significantly higher compared to the controls, respectively. However, only *S. riobrave* (355) had significantly higher mortality than the control for the pupae of *H. zea*. For the pupae of *C. includens*, except for *H. bacteriophora (HP88)*, *S. rarum (17c+e)*, and *H. floridensis* (K22), the mortality of the other seven strains was significantly higher than the control. Subsequently, *S. carpocapsae* (All) and *S. riobrave* (7–12) were chosen for efficacy testing in the field with an adjuvant 0.066% Southern Ag Surfactant (SAg Surfactant).

In field experiments, the SAg Surfactant treatment significantly increased the mortality and EPN infection for *S. carpocapsae* (All) on first instars of *H. zea* in corn plant whorls. On soybean plants, with the SAg Surfactant, *S. carpocapsae* (All) was more effective than *S. riobrave* (7–12) on fifth instars of *C. includens*. This study indicates that EPNs can control *H. zea* and *C. includens*, and SAg Surfactant can enhance EPN efficacy.

In the southern United States, *Helicoverpa zea* (Boddie) and *Chrysodeixis includens* (Walker) (Lepidoptera: Noctuidae) are economically important crop pests. *H. zea*, also known as the corn earworm, is a highly polyphagous pest that can cause damage to a wide range of agricultural crops, including corn (*Zea mays* L.), cotton (*Gossypium hirsutum* L.), and soybean [*Glycine max* (L.) Merrill] (Luttrell and Jackson, 2012; Swenson et al., 2013). While corn is a preferred host for *H. zea* (Lincoln and Isely, 1947; Hardwick, 1965), the insect also infests other crops, such as soybeans (Suits et al., 2017), when corn crops age past peak season. *C. includens*, commonly referred to as soybean looper, causes damage to vegetable and field crops, including soybean, cotton, alfalfa, sweet potato, and clovers (Hensley et al. 1964). In the Mississippi Delta region, *C. includens* was the most common Plusiinae species in soybean, particularly during the late season (July to September) (Jost and Pitre, 2002; Allen et al., 2021).

Bt corn and Bt soybean varieties have been approved for commercial planting by the U.S. Environmental Protection Agency (EPA) since 1995 and 2010, respectively (U.S. EPA, 2018), and initially provided effective control of targeted pests. However, many insect pests have developed resistance to these Bt crops, thereby reducing their effectiveness (Tabashnik and Carrière, 2017). While the frequency of Cry1Ac resistance alleles remains low and stable in *C. includens* (Horikoshi et al., 2021), *H. zea* has developed resistance to the Cry1A and Cry2A toxins found in Bt corn and cotton, and the efficacy has been significantly reduced in the field (Tabashnik and Carrière, 2017; Reisig and Kurtz, 2018; Yang et al., 2019; 2020). Therefore, there is a need to explore other control measures, such as biological control, to prolong the usefulness of Bt varieties (Hoffmann et al, 2014).

Entomopathogenic nematodes (EPNs) in the genera *Steinernema* and *Heterorhabditis* are important agents for the biological control of many insect pests. The infective juveniles (IJs) of EPNs release symbiotic bacteria in insect cavities and kill infested insects within 48 hr (Poinar, Jr., 1990). EPNs have been effectively used to control a variety of underground and some aboveground insect pests (Kaya et al., 2006; Lacey and Georgis, 2012; Shapiro-Ilan et al., 2017). However, the effectiveness of EPNs for aboveground applications can be limited due to issues such as desiccation (Glazer, 1992; Patel et al., 1997; Navaneethan et al., 2010) and ultraviolet radiation (Gaugler and Boush, 1978; Gaugler et al., 1992). To overcome these problems, several kinds of spray adjuvants have been tried to enhance the performance of EPNs against aboveground insect pests, including antidesiccants, humectants, surfactants, oil base solvents, or their combinations (Webster and Bronskill, 1968; Glazer, 1992; Macvean et al., 1982; Glazer et al., 1992; Noodidum et al., 2016; Portman et al., 2016; Schroer and Ehlers, 2005; Schroer et al, 2005; Shapiro-Ilan et al., 2010).

Previous studies have reported the effective use of EPN *S. riobrave* for the control of prepupae and pupae of *H. zea* underground (Cabanillas and Raulston, 1995, 1996; Feaster and Steinkraus, 1996). Moreover, *S. carpocapsae* has been shown to be effective in controlling the *H. zea* larvae aboveground (Bong and Sikorowski, 1983; Bong, 1986; Purcell et al., 1992). However, many of the better results for both underground and aboveground applications came from very high doses of EPNs. To our knowledge, no reports are available to indicate the pathogenicity or virulence of EPNs to *C. includens*. The 10 strains of *Steinernema* and *Heterorhabditis* species used in this study were from the EPN culture collection of USDA-ARS at Byron, Georgia. All 10 strains or some of these strains were evaluated against many insect pests, such as citrus root weevil, *Diaprepes abbreviatus* (L.) (Coleoptera: Curculionidae) (Shapiro et al., 1999); lesser peachtree borer, *Synanthedon pictipes* (Grote & Robinson) (Lepidoptera: Sesiidae) (Shapiro-Ilan et al., 2010); sugarbeet wireworm, *Limonius californicus* (Mannerheim) (Coleoptera: Elateridae) (Sandhi et al., 2020); sweetpotato whitefly, *Bemisia tabaci* Middle East-Asia Minor 1 (Hemiptera: Aleyrodidae) (Liet al., 2021). The objectives of this study were to (i) evaluate the virulence and efficacy of several EPN species against *H. zea* and C. *includens,* and (ii) examine the value of adding a commonly used adjuvant under field conditions.

## Materials and Methods

### Insect Rearing

The *H. zea* colony at the USDA ARS Southern Insect Management Research Unit (USDA ARS SIMRU) in Stoneville, Mississippi was initiated in 1971. The *C. includens* colony maintained at USDA ARS SIMRU was initially purchased from Benzon Research (Carlisle, Pennsylvania)*.* Both colonies are maintained routinely at 27°C, 14:10 light: dark photoperiod, and 70%–80% RH in environmental chambers (Percival Scientific, Perry, Iowa, USA) using the ARS soybean and wheat germ-based diet developed for *Heliothis virescens* (Blanco et al., 2009a, 2009b).

### EPN Rearing

Originally, 10 EPN strains were obtained from the USDA-ARS EPN Laboratory in Byron, Georgia. They were strains of seven species: *H. bacteriophora* [HP88 and VS strains (HbHP88 and HbHPVS)], *H. floridensis* [K22 strain (HfK22)], Hgkesha [kesha strain (Hgkesha)], *S. carpocapsae* [All and Cxrd strains (ScAll and ScCxrd)], *S. feltiae* [SN strain (SfSN)], *S. rarum* [17c+e strain (Sr17c+e)], and *S. riobrave* [355 and 7–12 strains (Sr355 and Sr7-12)]. The EPNs were then reared in the last instars of greater wax moth, *Galleria mellonella* L. (Lepidoptera: Pyralidae) (Josh's Frogs, Owosso, Michigan). The waxworms were inoculated at a ratio of 10 IJs per host. In a 100 x 15 mm petri dish containing a single layer of VWR filter paper, one mL of EPN suspension at a rate of 100 IJ/mL was pipetted along with 10 hosts. The petri dishes were then covered with a black plastic bag to protect them from light and incubated at room temperature (~22°C). After the hosts died from EPN infection in approximately 48 hr, the cadavers were moved to White traps (Kaya and Stock, 1997), which were also held at room temperature and covered with a black plastic bag. The White traps were checked every day for the emergence of IJs, which were collected and stored in tissue culture flasks in a 13°C refrigerator.

The day before each experiment, IJs were counted under a dissecting microscope using counting slides (Chalex, LLC, Park City, Utah, USA), and the suspensions of desired EPN rates were prepared from the counted source. For adjuvant treatments, the adjuvant was initially pipetted into 2-mL tubes but not added to the treatment containers until the day of the experiment. A volume of 1 mL of water was taken out from the treatment containers and combined with the adjuvants in the 2-mL tubes. The mixtures were then thoroughly mixed using a pipette, and then were poured back into the treatment containers, followed by another round of mixing.

### Laboratory Experiments

#### Effect of 10 EPN strains on eggs of *H. zea* and *C. includens*

Adults of *H. zea* or *C. includens* were placed in 3.78-L cardboard cartons with a source of 5% sugar water and covered with brown paper towels on the tops of cartons to collect eggs. Eggs used in the experiment were less than 24 hr old. Under a microscope, any dead (deflated) eggs were removed, and whole eggs were circled with a pencil for accessible location during experiments. Because egg fertilization could not be observed in advance, comparisons were made between unfertilized eggs from containers with unmated females of *H. zea*, and eggs included fertilized and unfertilized eggs collected from containers with both males and females of *H. zea*. For *C. includens*, only eggs from containers with both males and females of *C. includens* were tested because only a small number of unfertilized eggs were laid by unmated females. Each EPN strain was replicated three times with 10 host eggs per replication, and the entire experiment was repeated once. Ten marked eggs were placed in a 60 x 15 mm petri dish with a single layer of VWR filter paper with a piece of diet as a food source for any hatched larvae. Then, 1 mL of EPN suspension at a rate of 100 IJs/mL was pipetted into each petri dish. Control petri dishes received 1 mL of water. The petri dishes were then placed into a dark incubator set at 25°C, and a water reservoir was placed in the incubator to keep the relative humidity (RH) not lower than 85% for the duration of the experiment. Observations were made three times daily from 8:00 AM to 5:00 PM during the peak of the larvae hatching period, and newly hatched larvae were removed when observed. All unhatched eggs were dissected under a dissecting microscope to check for the presence of EPNs 7 d after exposure to EPNs. The number of eggs with EPNs present divided by the total number of eggs was used as the EPN infection and is reported in the result table.

#### Effect of 10 EPN strains on first to fourth instars, fifth instars, and pupae of *H. zea* and *C. includens*

As with the eggs of both pests, all 10 EPN strains were tested for efficacy against *H. zea* and *C. includens* at young larvae (first to fourth instars), mature larvae (fifth instars), and pupae. For the young larva group, the larvae used were late first instars at the beginning of the experiment, and the survivors were fourth instars at the end of the experiment. Each EPN strain was replicated three times for each insect group with 10 insects per replication, and the entire experiment was repeated once. A single insect was placed in a 60 x 15mm petri dish lined with a single layer of VWR filter paper and provided with a piece of diet as a food source. One mL of EPN suspension at a rate of 100 IJ/mL was pipetted into each petri dish, while control dishes received 1 mL of water. The petri dishes were then placed into a dark incubator set at 25°C, and a water reservoir was placed in the incubator to keep the RH not lower than 85% for the duration of the experiment. Dead larvae and pupae were recorded daily until young larvae developed to fourth instars on day 7, mature larvae developed into pupae on day 7, and pupa emerged to adults (7 d for *C. includens* and 12 d for *H. zea*). The number of dead larvae divided by the total number of larvae was used to calculate mortality reported in the result table.

### Field Experiments

#### General design for field experiments with two EPN strains: ScAll and Sr7-12

Field experiments with two EPN strains, ScAll and Sr7-12, were run to measure the efficacy of these EPNs at the ARS research farm located in Leland, Mississippi. Experiments were conducted in corn plantings (variety: DKC67-44, VT2P, DEKALB brand) and soybean plantings (variety: AG46x6, Asgrow brand). The adjuvant used in field experiments was 0.066% Southern Ag Surfactant for Herbicides (SAg Surfactant, Non-Ionic, containing active ingredients such as alkyl aryl polyoxyethylene glycol and other ethoxylated derivatives). All EPN treatments and controls were replicated four times, and each replication consisted of five randomly selected connected corn or soybean plants. The area for five corn or soybean plants in each replication was about 2,500 cm^2^. The distance between any two replications was > 4 m. Sixty mL pump sprayers (Crafter's Square) were used for application. Field temperature and RH were recorded for the period of application, from approximately 7:00 AM to 10:00 AM, using a digital hygrometer and thermometer (AcuRite band), and the daily minimum and maximum temperatures and RH were obtained from the local weather station (Burdett-KMSLELAN2), which was 0.8 km from the experiment blocks. After 24 hr, the corn or soybean plants used for the experiment were cut and carefully searched for the larvae of *H. zea or C. includens*, then placed individually in diet cups, incubated at room temperature (~22°C) and observed daily for mortality up to 72 hr. Any dead larvae were dissected under a dissecting microscope for the presence of EPNs. Further information for specific individual experiments is given below.

To prevent dead insects from decaying, dropping to the ground, or being lost to predators or other causes, insects were collected after 24-ht exposure to EPNs in field experiments, even though some live EPNs were still present on the plants after 24 hr. Because EPNs are sensitive to UV radiation and desiccation (Gaugler and Boush, 1978; Glazer, 1992; Patel et al., 1997), the dates for running the experiments were chosen to be as favorable as possible for EPNs, such as days with rain the day before the experiment or partial to full clouds on the day of the experiment. Experiments were started in the early morning when there was dew on the plants.

#### EPN strain ScAll with an adjuvant against *H. zea* larvae

In a corn field, EPN strain ScAll with SAg Surfactant was assessed against laboratory-reared late first instars of *H. zea* in a large block of corn plants on June 17, 2022, and June 27, 2022. Larvae were placed in corn center whorls, five larvae per plant and 25 larvae per replication. Each EPN replication received 10-mL EPN suspension, while each control replication received a 10-mL solution that did not contain any EPNs. Spraying was aimed at the centers of corn whorls.

The corn field Exp. #1 was run on June 17, 2022, when the corn plants were 6 wk old, approximately 1.1 m tall, and had 10 leaves per plant. The two EPN treatments were (i) ScAll suspended and (ii) ScAll suspended with SAg Surfactant, each applied at a rate of 20 IJs/cm^2^. The two controls were (i) water only and (ii) SAg Surfactant solution.

The Cornfield Exp. #2 was run on June 27, 2022 and was a repeat of the cornfield Exp. #1 except for the EPN rate, which was doubled to 40 IJs/cm^2^. In this experiment, the corn plants were seven and a half weeks old, approximately 1.3 m tall, and had 12 leaves for each plant.

#### Two EPN strains with an adjuvant against *C. includens* larvae

In a soybean field, two EPN strains, ScAll and Sr7-12 with SAg Surfactant, were tested against laboratory-reared fifth instars of *C. includens* in two large blocks of soybean plants on August 3, 2022 (soybean field Exp. #1), and September 8, 2022 (soybean field Exp. #2). Five laboratory-reared fifth instars of *C. includens* were prepared for each soybean plant. Because the leaves of the five soybean plants overlapped each other, so 25 *C. includens* larvae were placed on the leaves of five soybean plants randomly. Then, each EPN replication received a 20-mL EPN suspension, while each control replication received a 20-mL solution that did not contain any EPNs. The spray method was to tilt the sprayer back about 45° to target the undersides of the leaves specifically. In fact, the top surfaces of the leaves also received the suspension or solution at the same time. A second round of 40 mL water was applied to each replication 2 hr after the first spray to extend the wetting period and promote EPN survival.

The soybean field Exp. #1 was run on August 3, 2022, when soybean plants were 9 wk old and 0.5 m tall at the full pod stage. This experiment had two EPN treatments: (i) ScAll suspension with SAg Surfactant, and (ii) Sr7-12 suspension with SAg Surfactant, both applied at 40 IJs/cm^2^. The control was the SAg Surfactant solution.

The soybean field Exp. #2 was run on September 8, 2022 and was a repeat of the soybean field Exp. #1 but with a lower EPN rate of 10 IJs/cm^2^. In this experiment, the soybean plants were 8 wk old and approximately 0.4 m tall at the beginning of the pod formation stage. The soybean Exp. #2 was run in a second large block, which was next to the block for Exp. #1.

Because many *C. includens* larvae were missing or attacked by natural enemies during the field soybean Exp. #1 on August 3, 2022, a 1.5 x 1.0-m garden netting bag (0.8 mm x 1 mm mesh hole, Meuallikit brand, China) was used to cover each replication for the soybean field Exp. #2 on September 8, 2022.

## Data Analysis

Original percentages were normalized using arcsine square root transformation before performing the analysis of variance (ANOVA) (Richter and Fuxa, 1990). Treatment means were separated in JMP version 15.0.0 using Tukey HSD (α < 0.05). Untransformed treatment means are presented in the tables.

## Results

### Laboratory Experiments

#### Effect of ten EPN strains on eggs of *H. zea* and *C. includens*

**Figure 1:**
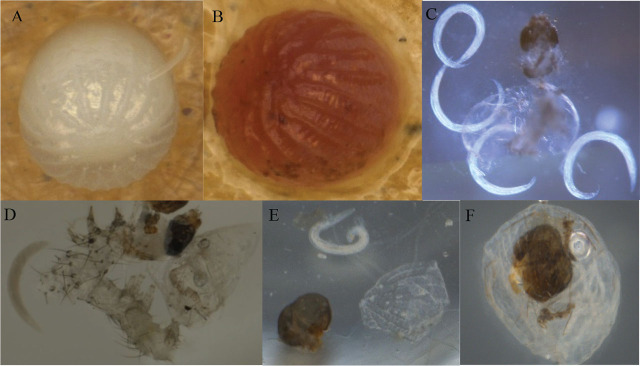
EPN infection on eggs of *Helicoverpa zea* and *Chrysodeixis includens*. (A) An infectious juvenile of *Steinernema carpocapsae* (all strain) was infecting an egg of *H. zea* with part of its body inside the egg. (B) An infected egg of *H. zea* by *Heterorhabditis georgiana* (kesha strain), from which 50 progeny juveniles were produced. (C) A larva of *H. zea* killed inside an egg by *H. floridensis* (K22) with young juveniles inside four adult female nematodes. (D) A larva of *C. includens* killed inside an egg by a juvenile of *S. feltiae* (SN). (E) A head capsule of a *C. includens* larva left after a juvenile of *S. feltiae* (SN) developed inside the egg. (F) A head capsule of a *C. includens* larva inside an egg with newly emerged juveniles of *S. feltiae* (SN).

**Figure 2:**
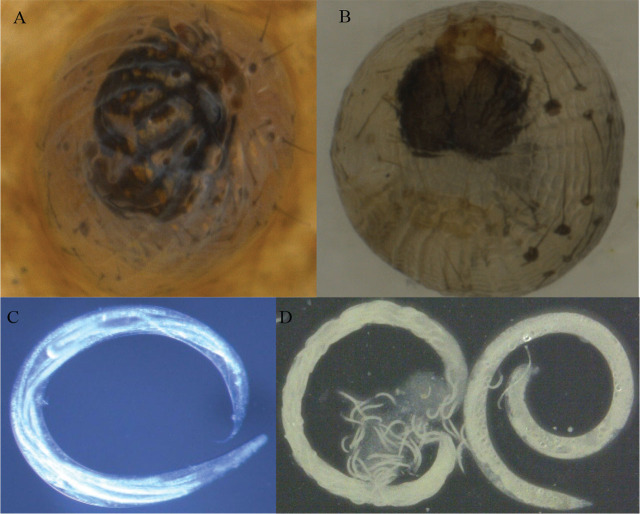
Larvae of (A) *Helicoverpa zea* and (B) *Chrysodeixis includens* inside eggs where larvae biting the eggshells and the protruding hairs of larvae were visible. (C) An adult of *Heterorhabditis floridensis* (K22 strain) from an *H. zea* egg with a few juveniles inside its body, and (D) two adults of *Steinernema carpocapsae* (all strain) from a fifth instar of *C. includens* with many juveniles inside one adult and eggs inside another.

The EPN infection as the unhatched eggs with EPN present after dissection divided by the total number of eggs is reported in Table 1. Data showed that EPNs could infect the eggs of *H. zea* or *C. includens* in the laboratory, but the infection was low, less than 11.7%. Figure 1A shows an infective juvenile invading an egg of *H. zea*. Figure 1B, the red egg of *H. zea*, indicates the typical signs of infection by heterorhabditid nematodes; this egg was infected with *Hgkesha*. In most infected eggs, only a single EPN was found (Figs. 1D and 1E), but sometimes multiple EPNs were found in a single egg (Fig. 1C) before emergence of EPN progeny. As EPNs developed in the eggs, sometimes the eggs expanded and then shrinked, at which points the EPN progeny were found inside the eggs (Fig. 1F). The infection to the unfertilized eggs of *H. zea* indicated that EPNs can infect seamless eggs. In addition, as the insect larvae developed and moved within the eggs, eggshells were sometimes damaged (Figs. 2A and 2B), and such points of mechanical damage might allow EPNs to enter eggs and kill larvae before they hatched. However, the number of young progeny found inside EPN female adults that developed in eggs was much lower than that developed in larvae (Figs. 2C and 2D). In this experiment, the maximum number of progeny IJs found in an egg of *H. zea* and an egg of *C. includens* were 50 IJs for Hgkesha and 36 IJs for ScCxrd, respectively. For comparison, five individual fifth instars of *H. zea* or *C. includens* were checked using White traps. The maximum number of IJs recovered from one larva of *H. zea* was 75,500 IJs for Hgkesha, and from one larva of *C. includens* was 72,000 IJs for ScCxrd. When new hosts were infected with the IJs that had developed in eggs of *H. zea* or *C. includens*, healthy progeny were produced normally.

**Table 1:** Entomopathogenic nematodes infection (% ± SEM)) on eggs of *Helicoverpa zea* and *Chrysodeixis includens* in the laboratory (25°C, 85% RH).

**EPN strain**	***H. zea***		***C. includens***
	
	**Unfertilized eggs^a^**	**Mixed eggs**	**Mixed eggs**
*Heterorhabditis bacteriophora* (HP88)	0.0 ± 0.0 a	5.0 ± 3.4 ab	6.7 ± 3.3 a
*Heterorhabditis bacteriophora* (VS)	1.7 ± 1.7 a	1.7 ± 1.7 ab	0.0 ± 0.0 a
*Heterorhabditis floridensis* (K22)	6.7 ± 3.3 a	10.0 ± 6.3 ab	0.0 ± 0.0 a
*Heterorhabditis georgiana* (kesha)	0.0 ± 0.0 a	11.7 ± 4.0 a	0.0 ± 0.0 a
*Steinernema carpocapsae* (All)	1.7 ± 1.7 a	0.0 ± 0.0 b	1.7 ± 1.7 a
*Steinernema carpocapsae* (Cxrd)	1.7 ± 1.7 a	3.3 ± 1.7 ab	1.7 ± 1.7 a
*Steinernema feltiae* (SN)	0.0 ± 0.0 a	3.3 ± 2.1 ab	6.7 ± 4.9 a
*Steinernema rarum* (17c+e)	0.0 ± 0.0 a	1.7 ± 1.7 ab	0.0 ± 0.0 a
*Steinernema riobrave* (355)	1.7 ± 1.7 a	6.7 ± 2.1 ab	0.0 ± 0.0 a
*Steinernema riobrave* (7–12)	1.7 ± 1.7 a	0.0 ± 0.0 b	3.3 ± 3.3 a
Water	0.0 ± 0.0 a	0.0 ± 0.0 b	0.0 ± 0.0 a

*F*-value (10, 55)	1.53	2.64	1.64
*P*-value	0.1534	0.0104	0.1193

Mean values within a column followed by the same letter are not significantly different at *P* > 0.05 (Tukey's HSD test).

a Unfertilized eggs were collected from the container with unmated females. Mixed eggs included fertilized and unfertilized eggs were collected from the container with both males and females.

#### Effect of 10 EPN strains on first to fourth instars, fifth instars, and pupae of *H. zea* and *C. includens*

**Figure 3:**
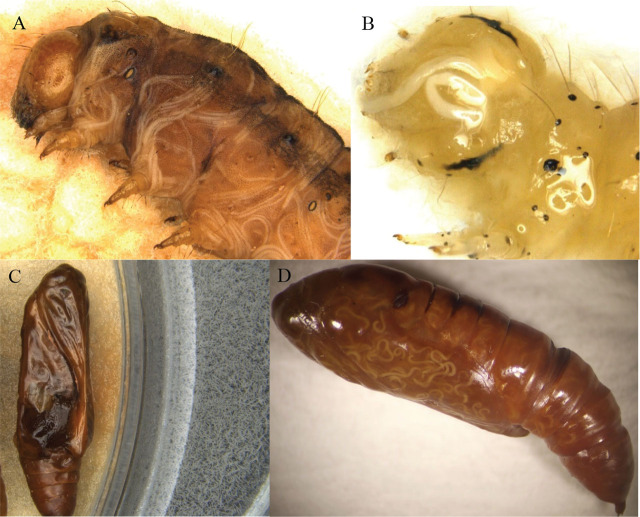
EPN infection of larvae and pupae of *Helicoverpa zea* and *Chrysodeixis includens*. (A) Adults of *Steinernema rarum* (17c+e strain) inside a larva of *H. zea*. (B) Large adults of *S. carpocapsae* (all) inside the head of *C. includens* larva. (C) Juveniles of *S. riobrave* (7–12) from a pupa of *H. zea* in a White trap. (D) Adults of *Heterorhabditis georgiana* (kesha strain) inside a pupa of *Chrysodeixis includens*.

In this study, the mortality caused by 10 EPN strains in 7 d was significantly higher for the first to fourth instars of *H. zea* compared to the control, and as was the fifth instars of *H. zea*. Similarly, for the first to fourth and fifth instars of *C. includens*, the mortality was significantly higher compared to the controls, respectively (Table 2). The mortality caused by these 10 EPN strains did not show significant variation for the first to fourth instars of *H. zea* (ranging from 98.3% to 100.0%). For the fifth instars of *H. zea*, the mortality caused by ScAll and Sr7-12 (95.0% and 93.3%) was significantly higher than that of Sr17c+e (58.3%), but there was no significant difference from the other seven EPN strains. For the first to fourth instars of *C. includens*, the mortality caused by seven EPN strains (Sr7-12, Hgkesha, HfK22, Sr355, ScAll, ScCxrd, and HbHp88, ranging from 80.0%–90.0%) was significantly higher than that of SfSN (40.0%), but there was no significant difference from HbVS and Sr17c+e (72.6% and 71.7%). Additionally, there was no significant difference in the mortality among the 10 EPN strains for the fifth instars of *C. includens* (ranging from 88.3% to 100.0%). Figures 3A and 3B show EPNs that have developed into adults within the larvae of *H. zea* and *C. includens*.

**Table 2:** Mortality (% ± SEM) of first to fourth instars, fifth instars, and pupae of *Helicoverpa. zea* and *Chrysodeixis includens* caused by 10 entomopathogenic nematode strains in the laboratory (25°C, 85% RH).

**EPN strains**	***H. zea***			***C. includens***			***H. zea* and *C. includens***
		
	**First-fourth instars**	**Fifth instars**	**Pupae**	**First-fourth instars**	**Fifth instars**	**Pupae**	**Avg of first-fourthand fifth instars^a^**
*Heterorhabditis bacteriophora* (HP88)	100.0 ± 0.0 a	81.7 ± 6.0 ab	6.7 ± 4.9 ab	80.0 ± 7.3 a	96.7 ± 2.1 a	33.3 ± 11.7 abc	89.6
*Heterorhabditis bacteriophora* (VS)	98.3 ± 1.7 a	81.7 ± 4.8 ab	15.0 ± 4.3 ab	72.6 ± 5.4 ab	93.3 ± 4.9 a	40.0 ± 11.8 ab	86.5
*Heterorhabditis floridensis* (K22)	98.3 ± 1.7 a	85.0 ± 7.6 ab	21.7 ± 8.7 ab	88.3 ± 3.1 a	100.0 ± 0.0 a	30.0 ± 8.9 abc	92.9 (top 5)
*Heterorhabditis georgiana* (kesha)	100.0 ± 0.0 a	83.3 ± 4.2 ab	13.3 ± 4.2 ab	90.0 ± 4.5 a	95.0 ± 3.4 a	45.0 ± 5.0 ab	92.0 (top 5)
*Steinernema carpocapsae* (All)	100.0 ± 0.0 a	95.0 ± 3.4 a	20.0 ± 6.8 ab	85.0 ± 2.2 a	98.3 ± 1.7 a	41.7 ± 9.5 ab	94.6 (top 5)
*Steinernema carpocapsae* (Cxrd)	100.0 ± 0.0 a	85.0 ± 8.1 ab	21.7 ± 6.0 ab	80.0 ± 8.6 a	98.3 ± 1.7 a	36.7 ± 12.3 ab	90.8
*Steinernema feltiae* (SN)	100.0 ± 0.0 a	76.7 ± 6.7 ab	5.0 ± 2.2 ab	40.0 ± 5.8 b	95.0 ± 3.4 a	18.3 ± 6.0 bc	77.9
*Steinernema rarum* (17c+e)	100.0 ± 0.0 a	58.3 ± 13.8 b	13.3 ± 5.6 ab	71.7 ± 7.4 ab	88.3 ± 7.9 a	26.7 ± 4.9 abc	79.6
*Steinernema riobrave* (355)	100.0 ± 0.0 a	88.3 ± 5.4 ab	21.7 ± 6.5 a	86.7 ± 6.1 a	100.0 ± 0.0 a	61.7 ± 7.0 a	93.8 (top 5)
*Steinernema riobrave* (7–12)	100.0 ± 0.0 a	93.3 ± 4.9 a	15.0 ± 4.3 ab	90.0 ± 5.2 a	98.3 ± 1.7 a	63. 3 ± 4.2 a	95.4 (top 5)
Water	3.3 ± 3.3 b	0.0 ± 0.0 c	0.0 ± 0.0 b	0.0 ± 0.0 c	0.0 ± 0.0 b	1.7 ± 1.7 c	0

*F*-Value (10, 55)	187.99	15.81	2.42	18.4	41.62	4.99	
*P*-Value	<.0001	<.0001	0.0184	<.0001	<.0001	<.0001	

Mean values within a column followed by the same letter are not significantly different at *P* > 0.05 (Tukey's HSD test).

a The average mortality (%) of 1^st^–4^th^ and 5^th^ instars of *H. zea* and *C. includens* were not included in ANOVA and Tukey's HSD tests.

For the pupae of *H. zea*, only Sr355 (21.7%) had significantly higher mortality compared to the control. However, for the pupae of *C. includens*, six EPN strains (Sr7-12, Sr355, Hgkesha, ScAll, HbVS, and Scxrd) had significantly higher mortality, ranging from 36.7% to 63.3% in comparison to the control. Figure 3C shows the progeny IJs from a pupa of *H. zea* in a White trap, while Figure 3D shows EPNs that have developed into adults within a pupa of *C. includens*.

Based on the average mortality of first to fourth instars and fifth instars for both *H. zea* and *C. includens* (Table 2), the top five strains were found to be Sr7-12, ScAll, Sr355, HfK22, and Hgkesha. After taking into consideration their rearing performance and information from reference papers, the final selection for the field experiments was narrowed down to two EPN species, ScAll and Sr7-12. This conclusion was also made based on the fact that *S. carpocapsae* and *S. riobrave* are commercially available, whereas *H. flordensis* and Hgkesha are not.

### Field Experiments

#### EPN strain ScAll with an adjuvant against *H. zea* larvae

**Figure 4:**
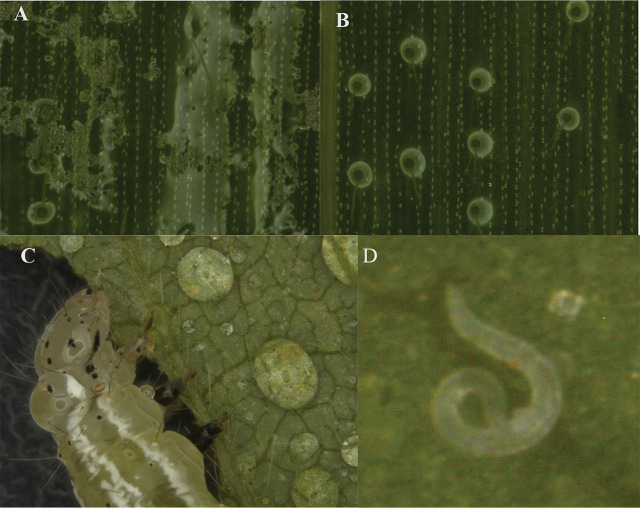
EPN suspension with and without an adjuvant. (A) Without an adjuvant, suspension was distributed unevenly on a corn leaf; air bubbles were visible. (B) With SAg Surfactant, suspension was distributed evenly across the corn leaf, which may help nematodes to find hosts and hide in pores on a corn leaf. (C) Without an adjuvant, many EPNs were trapped in water droplets, including on the leaf and insect. (D) With SAg Surfactant, a juvenile was able to search and move around more freely, allowing it to attempt to hide in a small leaf gap when the leaf was getting dry.

In this experiment, the EPN strain ScAll with the adjuvant SAg Surfactant was assessed in a corn field against laboratory-reared late first instars of *H. zea* on June 17 and June 27, 2022, each date being a separate experiment. Figure 4A shows that without the SAg Surfactant, EPN suspension was unevenly distributed on the corn leaves. In contrast, with the SAg Surfactant, EPN suspension was evenly distributed on the corn leaves (Fig. 4B).

In Exp. #1 (on June 17, 2022), EPNs were applied at 20 IJs/cm^2^ and 2 ml/plant, either with or without the SAg Surfactant. Thirty minutes of heavy rain (precipitation accumulated 0.43 cm) occurred 5 hr after spraying. Our results showed that including SAg Surfactant did not increase larval mortality (78.2% with SAg Surfactant and 69.3% without SAg Surfactant) and EPN infection (35.0% with SAg Surfactant and 24.2% without SAg Surfactant) (Table 3). However, the heavy rain did not wash away all the EPNs, and some EPNs were still visible on the plants the next day and even 7 d later.

**Table 3:** Effect of *Steinernema carpocapsae* (all strain) with an adjuvant on first instars of *Helicoverpa zea* in corn plants in a corn field (2022, Leland, Mississippi).

**Field exp., #** **Experiment date** **Temperature** **RH** **Weather condition**	**EPN Rate** **Plant Age** **Plant high**	**Treatment**	**# of Insects**	**% Mortality ± SEM**	**% EPN Infection ± SEM**
Field exp, #1	20 IJs/cm^2^	*S. carpocapsae* (All) + SAg^	63	78.2 ± 9.2 a	35.0 ± 10.3 a
June 17, 2022					
24(23–35) °C^a^	6 wk	*S. carpocapsae* (All)	56	69.3 ± 7.8 a	24.2 ± 10.2 a
82(62–99) %RH	1.1 m	Water + SAg	64	22.0 ± 4.6 b	0.0 ± 0.0 b
30 min heavy rain		Water	53	28.0 ± 8.1 b	0.0 ± 0.0 b
				F(3, 12)=20.66	F(3, 12)=10.95
				*P* < 0.0001	*P* < 0.0009
Field exp., #2	40 IJs/cm^2^	*S. carpocapsae* (All) + SAg	80	87.7 ± 1.1 a	66.4 ± 4.7 a
June 27, 2022					
22(19–30) °C	7.5 wk	*S. carpocapsae* (All)	58	70.4 ± 3.6 b	42.0 ± 2.5 b
83(58–94) %RH	1.3 m	Water + SAg	65	18.7 ± 4.5 c	0.0 ± 0.0 c
Partly Cloudy		Water	74	21.6 ± 3.1 c	0.0 ± 0.0 c
				F(3, 12)=77.26	F(3, 12)=294.09
				*P* < 0.0001	*P* < 0.0001)

Mean values within a column followed by the same letter are not significantly different at *P* > 0.05 (Tukey's HSD test).

a Numbers outside parentheses were the temperature or RH at the beginning of spaying, numbers inside parentheses were the lowest-highest temperature or RH during 24 hr.

^SAg= 0.066% SAg Surfactant.

Exp. #2 (on June 27, 2022) was intended to be a repeat of Exp. #1. EPNs were applied at a higher rate (40 IJs/cm^2^ and 2ml/plant). In this experiment, SAg Surfactant significantly increased the larval mortality (87.7% with SAg Surfactant and 70.4% without SAg Surfactant), and the EPN infection was also significantly increased (66.4% with SAg Surfactant and 42.0% without SAg Surfactant) compared to the same EPN strain without SAg surfactant (Table 3).

#### Two EPN strains with an adjuvant against *C. includens* larvae

Following the outcomes of the experiment conducted in the corn field on June 27, 2022, it was evident that the addition of SAg Surfactant led to a significant increase in larval mortality and EPN infection when compared to no addition of SAg Surfactant. Subsequently, the EPN strain ScAll, both with and without SAg Surfactant, was sprayed onto soybean leaves to assess the activities of EPNs. Figures 4C and 4D show the EPN suspension with and without SAg Surfactant on soybean leaves. The EPN suspension without SAg Surfactant was distributed on the leaves in the form of water droplets, while the EPN suspension with SAg Surfactant was evenly distributed on the leaves. This was consistent with what was seen on the corn leaves mentioned above in Figures 4A and 4B. Therefore, to reduce the amount of work and materials, only the treatments with SAg Surfactant were conducted to compare the effects of two EPN strains on *C. includens* larvae in this experiment.

In Exp. #1 (August 3, 2022), ScAll caused significantly higher larval mortality and EPN infection than Sr7-12 did (mortality, 92.9% for ScAll and 25.2% for Sr7-12; infection 37.1% for ScAll and 0.0% for Sr7-12) (Table 4). In the Exp. #2 (a repetition of Exp. #1) on September 8, 2022, the results were like those in Exp. #1. ScAll caused significantly higher mortality and EPN infection than Sr7-12 did (mortality, 23.7% for ScAll and 25.2% for Sr7-12; infection 7.1% for ScAll and 0.0% for Sr7-12). Even though no EPNs were found inside the dead *C. includens* larvae treated with Sr7-12 in either experiment, it was still possible that EPNs killed the dead insects.

**Table 4:** Effect of *Steinernema riobrave* (7–12 strain) and *Steinernema carpocapsae* (all strain) with an adjuvant on fifth instars of *Chrysodeixis includens* on soybean plants in soybean fields (2022, Leland, Mississippi).

**Experiment date** **Temperature** **RH** **Weather condition**	**EPN rate** **Plant age** **Plant high**	**Treatment**	**# of Insects**	**% Mortality ± SEM**	**% EPN Infection ± SEM**
Field exp., #1 August 3, 2022	40 IJs/cm^2^	*S. carpocapsae* (All) + SAg^	37	92.9 ± 4.7 a	37.1 ± 7.6 a
24(22–35) °C^a^	9 wk	*S. riobrave* (7–12) + SAg	51	25.2 ± 12.6 b	0.0 ± 0.0 b
88(60–97) %RH	0.5 m	Water + SAg	38	13.0 ± 7.2 b	0.0 ± 0.0 b
Most sunny				F(2, 9)=16.82	F(2, 9)=58.4
				P=0.0009	*P* < 0.0001
					
Field exp., #2 September 8, 2022	10 IJs/cm^2^	*S. carpocapsae* (All) + SAg	95	23.7 ± 4.8 a	7.1 ± 1.4 a
22(19–32) °C	8 wk	*S. riobrave* (7–12) + SAg	91	6.2 ± 1.3 b	0.0 ± 0.0 b
79(40–95) %RH	0.4 m	Water + SAg	84	1.09 ± 1.1 b	0.0 ± 0.0 b
Sunny				F(2, 9)=21.28	F(2, 9)=73.04
				P=0.0004	*P* < 0.0001

Mean values within a column followed by the same letter are not significantly different at *P* > 0.05 (Tukey's HSD test).

a Numbers outside parentheses were the temperature or RH at the beginning of spaying, numbers inside parentheses were the lowest-highest temperature or RH during 24 hr.

^SAg= 0.066% SAg Surfactant.

## Discussion

This study indicates that EPNs can control *H. zea* and *C. includens*, and that an adjuvant can enhance EPN efficacy when ScAll is used against *H. zea* larvae in corn plant whorls. The study is the first to demonstrate the effectiveness of EPNs against the soybean looper, *C. includens.* The results from field experiments indicate the potential of using EPNs to control *C. includens* effectively when the correct life stage and favorable weather conditions are present.

The high mortality observed in the larval stage of both *H. zea* and *C. includens* under laboratory conditions suggest that the larval stage is the most susceptible stage to EPNs. This susceptibility prompted the selection of larvae for the field experiments. The low infection on the eggs of *H. zea* and *C. includens* under laboratory conditions suggests that use of EPNs to control eggs of *H. zea* and *C. includens* under field conditions will be a challenge. However, it demonstrates the capacity of EPNs to infect and develop within eggs. Unlike the egg and larval stages of *H. zea*, and all stages of *C. includens* that inhabit aboveground habitats, the prepupa and pupa stages of *H. zea* inhabit underground, which is more favorable for EPN activities than aboveground. Cabanillas and Raulston (1996) showed that under field conditions, using *S. riobrave* and *S. carpocapsae* against prepupae and pupae of *H. zea* at a concentration of 200,000 IJs/m^2^, *S. riobrave* (95% parasitism) was more effective than *S. carpocapsae* (0% parasitism) at high field soil temperatures. In our laboratory experiments, for pupae of *H. zea*, among 10 strains of EPNs, only Sr355 showed a significantly higher mortality compared to the control. This makes S. *riobrave* an attractive candidate for biological control of underground prepupae and pupae of *H. zea.* However, more information from the 10 EPN strains is still needed to identify the most potent EPN strain among the 10 strains.

In field conditions, dew on plants is an intuitive indicator of field humidity. The more dew and the longer it lasts, the more conducive it is to the activity of EPNs. The whorls of corn leaves intertwine, which may help retain moisture. Also, the small pockets between the corn leaf collar and ligule, a membrane-like structure located at the junction of the leaf blade and leaf sheath where the leaf attaches to the stalk, could hold a thin layer of water that might be favorable for nematode activities. In our experiments in corn plants, more live EPNs were found in these small pockets than other areas the next day after application. Densely planted soybeans may be beneficial to the formation of dew, and the densely planted soybean leaves can shade each other, which may play a certain role in conserving moisture and blocking sunlight. When dew appears at night, those nematodes that survived during the daytime can become active again. In our field experiments on our soybean plants, some viable EPN was still found the next day after application. The dates for running the experiments were chosen days with rain the day before the experiment or partial to full clouds on the day of the experiment because there was more dew on plants and dew lasted longer in those days.

According to Gaugler and Boush (1978), the IJs of *S. carpocapsae* lose almost all their ability to infect hosts after 60 min of direct sunlight exposure. Since eggs, larvae, and pupae of *C. includens* are usually found on the underside of soybean leaves, spraying EPN suspension on the underside of leaves in the present study may promote the chances for EPN to find the hosts and also may reduce the exposure of EPNs to direct sunlight, thereby promoting EPN survival. Furthermore, this study showed that ScAll was more effective against *C. includens* on soybean plants than Sr7-12 under field conditions, which may be attributed to its desiccation tolerance and superior UV (Patel et al., 1997; Shapiro-Ilan et al., 2015). Baur (1995) showed that *S. carpocapsae* was the most promising EPN for use against the diamondback moth, *Plutella xylostella* (L.) (Lepidoptera: Plutellidae) because it had a lower LC50 than the other species tested and survived well under desiccating conditions on a leaf surface. Their results also showed that, overall, EPN survival and infectivity to *P. xylostella* larvae were lower for *S. riobrave* than for *S. carpocapsae*. This makes *S. carpocapsae* a potential candidate for biological control *C. includens* on foliage application.

Previous studies have reported the use of surfactants in aboveground applications. Schroer and Ehlers (2005) used EPN *S. carpocapsae* on cabbage leaves to control *P. xylostella* larvae. *S. carpocapsae* applied in water resulted in *P. xylostella* mortality of 40%, and the use of a formulation containing 0.3% of the surfactant Rimulgan and 0.3% of the polymer xanthan significantly increased mortality to 75%. Bong and Sikorowski (1983) and Bong (1986) used *S. carpocapsae* (=*Neoaplectana carpocapsae* Weiser) (DD136 strain) against *H. zea* [= *Heliothis zea* (Boddie)] in corn ears, resulting in significant mortality of *H. zea*. The EPNs used in these tests contained 0.01% Triton X-100 surfactant. In our study, SAg Surfactant did not significantly increase larval mortality or EPN infection when ScAll was used against *H. zea* in corn plant whorls on June 17, 2022. This is possibly due to heavy rain during the experiment. However, the June 27, 2022 results emphasize that the SAg Surfactant can enhance EPN efficacy compared to unadjuvanted EPN. The critical component of SAg Surfactants is polyalkylene glycol, a well-known synthetic lubricant (Millett, 1950). These polymers are often used not only as lubricants but also as surfactants in surface treatment applications. SAg Surfactant acts as a wetting agent, effectively reducing the surface tension of water. Adding wetting agents to liquid sprays promotes even distribution and adhesion to plant and insect surfaces. This, in turn, prevents the formation of isolated water droplets that may hinder EPNs from finding hosts and suitable hiding spots when surfaces are dry. Further investigation is needed to determine whether SAg Surfactants can enhance lubrication, potentially shortening the duration of infection and facilitating EPNs entry into the hosts.

In the present work, only two strains of EPNs, ScAll and Sr7-12, were tested in field conditions. There is a need to explore the other top-performing strains, Sr355, Hfk22, and Hgkesha, as well as additional adjuvants against the two target pests further. According to Grewal et al. (1994), in the temperature tolerance test, only *S. riobrave* among the 12 EPN strains was able to infect *G. mellonella* at 39°C. Shapiro-Ilan et al. (2014) reported that HfK22 has extremely high heat resistance and moderate cold resistance, and similar to Sr355, the HfK22 was capable of infecting a host up to 39°C. Regarding Hgkesha, though it generally possesses poor to moderate abilities in virulence and environmental tolerance relative to other EPNs for the range of hosts and conditions tested (Shapiro-Ilan et al., 2009), it works well in our laboratory tests and needs further evaluation in field conditions.
